# Inflammatory Myofibroblastic Tumor of the Urinary Bladder: An 11-Year Retrospective Study From a Single Center

**DOI:** 10.3389/fmed.2022.831952

**Published:** 2022-03-03

**Authors:** Can Chen, Mengjun Huang, Haiqing He, Shuiqing Wu, Mingke Liu, Jun He, Hongjing Zang, Ran Xu

**Affiliations:** ^1^Department of Urology, The Second Xiangya Hospital of Central South University, Changsha, China; ^2^Department of Pathology, The Second Xiangya Hospital of Central South University, Changsha, China

**Keywords:** inflammatory myofibroblastic tumor (IMT), bladder, retrospective study, treatment, diagnosis

## Abstract

**Purpose:**

To share our experience in the diagnosis and treatment of an inflammatory myofibroblastic tumor of the urinary bladder (IMTUB).

**Materials and Methods:**

A database searches in the pathology archives by using the term “inflammatory myofibroblastic tumor” and” bladder” in our hospital department of pathology from 2010 to 2021. Patient characteristics, clinical features, histopathological results, immunohistochemical staining results, and treatment outcomes were reviewed.

**Results:**

Fourteen cases of IMTUB were retrieved. The mean age was 44.7 ± 18.9 years (range 12–74). Nine (64.3%) of the patients presented with hematuria, followed by seven (50%) with odynuria, five (35.7%) with urgent urination, and one (7.1%) with dysuria. Ten (71.4%) of the patients were treated with partial cystectomy (PC), three (21.4%) with transurethral resection of bladder tumor (TURBT), and one (7.1%) with radical cystectomy (RC). Histopathologically, eight (57.1%) had a compact spindle cell pattern. Anaplastic lymphoma kinase (ALK) staining was positive in six (75%) of 8 cases. During a mean follow-up period of 43.9 ± 38 months (range 3–117), a patient had recurrence within half a month. Then, the patient was treated with further TURBT surgery and had no recurrence within 6 months. Thirteen of the patients had no local recurrence or distant metastasis.

**Conclusion:**

Inflammatory myofibroblastic tumor of the urinary bladder (IMTUB) is clinically rare and has a good prognosis. The disease is mainly treated with surgery to remove the tumor completely. It can easily be misdiagnosed as bladder urothelial carcinoma, leiomyosarcoma, or rhabdomyosarcoma, which may result in overtreatment and poor quality of life of patients.

## Introduction

An inflammatory myofibroblastic tumor (IMT) is a rare tumor made up of spindle cells with an associated inflammatory cell infiltrate (1). The pathogenesis and malignancy potential of the disease remain unclear (1, 2). The disease can occur anywhere in the body but is most commonly seen in the lungs, mesentery, and omentum (3, 4). In the genitourinary system, IMT is more likely to be found in the bladder. In previous literature, this disease has been reported in <1% of bladder tumors (5). Because of the low recurrence rate (only 4%), bladder-sparing treatment modalities, such as TURBT or partial cystectomy, are recommended (5, 6). Fourteen cases of IMTUB in our region were reviewed by retrospective analysis.

## Methods

A total of 14 patients diagnosed with IMTUB were recruited at Second Xiangya Hospital of Central South University in China from 2010 to 2021. Only IMTUB cases were included, and postoperative spindle cell nodule cases were excluded from our study. Each patient was treated primarily with surgery to remove the tumor and diagnosed by histopathological analysis. Immunohistochemical staining of anaplastic lymphoma kinase (ALK), S-100, desmin, smooth muscle actin (SMA), vimentin, cytokeratin (CK), CD34, CD68, CD117, Ki-67, and HMB-45 was performed to distinguish IMTUB from other tumors.

Clinical information included patient characteristics and tumor parameters. Patient characteristics, such as sex, age, presenting symptoms (including hematuria, odynuria, urgent urination, and dysuria), routine blood examination, routine urinalysis, preoperative urine culture, cystoscopy, abdominal computed tomography (CT), tumor parameter (tumor size in maximal dimension and tumor location in the urinary bladder), treatment and follow-up outcome, and histopathology and immunohistochemistry results were reviewed.

Histopathologically, IMTs can be categorized into three histopathological subtypes based on pathological morphology: the mucous/vascular type, the compact spindle cell type, and the hypocellular fibrous type (5, 7, 8). The mucous/vascular type features fasciitis, edema, or loose arrangement of plump cells in a mucinous stroma with prominent vessels. Inflammatory cells usually consist of more neutrophils and eosinophils and fewer plasma cells. The compact spindle cell type is mainly composed of proliferating spindle cells with bundles or layers. Large numbers of plasma cells and lymphocytes are typically mixed with spindle cells. The hypocellular fibrous type is similar to the fibromatosis type, but with vimineous rather than full spindle cells on a background of dense collagenous stroma, including sporadic plasma cells, eosinophils, and lymphocytes. Histopathologically, tumors can present with one type or a combination of two or three types (7, 8). In addition to the three histopathological types, other histopathological characteristics (such as the presence of necrosis, atypia, pleomorphism, abnormal mitosis, and mitotic figures) and a large number of inflammatory cells (such as lymphocytes, plasma cells, neutrophils, and eosinophils) were recorded. Depth of tumor invasion and all immunohistochemical results were recorded.

Because of the small number of patients, no statistical methods could be used. Informed consent was obtained from all the patients in our study, and this study was approved by the ethics committee of the Second Xiangya Hospital of Central South University.

## Results

### Clinical Features

A total of 14 patients, nine women and five men, were included, with a mean age of 44.7 ± 18.9 years (range 12–74). Nine (64.2%) of the patients complained of hematuria, six (42.9%) complained of odynuria, five (35.7%) complained of urgent urination, and one (7.1%) complained of dysuria. Abdominal CT examinations indicated space-occupying changes in the bladder. No hydronephrosis or urinary calculus was found, but ureter invasion by the tumor was suspected in 1 case on imaging examination. Five (35.7%) of the patients were admitted with anemia, with a mean hemoglobin level of 97.6 ± 13.7 g/dl (range 80–113). All the patients had normal serum creatinine levels at presentation. Interestingly, in case 3 and case 9, preoperative urine culture indicated *Enterobacter cloacae* infection, which has never been reported in previous literature, and the perioperative anti-infection effect was remarkable. This may be a predisposing factor for IMT because of chronic inflammation in the bladder. The other patients had no significant predisposing factors such as pregnancy, infection, and surgery. In addition, one 14-year-old boy presented with severe bladder irritation with systemic inflammation. Routine white blood cell count was 18.2*10^9^/L, NEUT% was 92.7%, PCT was 6.57 ng/ml, CRP was 366 mg/l, and ESR was 59 mm/h. Mean tumor size in maximal dimension was 33.9 ± 14.8 mm (range 13–70). Regarding tumor location in the urinary bladder, five of the patients (35.7%) had tumors on the right lateral wall, four (28.6%) had tumors on the anterior wall, three (21.4%) had tumors in the dome, one (7.1%) had a tumor on the left lateral wall, and one (7.1%) had a tumor on the anterosuperior wall. The important clinical features are summarized in Table 1.

**Table 1:** Clinical features, treatment and follow-up outcome of the 14 cases of IMTUB.

**Case (*N*)**	**Age (years)**	**Gender (M/F)**	**Symptoms**	**Location**	**Size (mm)**	**Treatment**	**Follow-up** **(months)**	**Recurrence** **or distant metastases**
1	41	M	Hematuria and urgent urination	Left lateral wall	27	PC	48	No
2	22	M	Urgent urination and odynuria	Anterior wall	70	PC	117	No
3	73	M	Urgent urination and odynuria	Right lateral wall	30	PC	96	No
4	62	F	Urgent urination	Right lateral wall	13	PC	100	No
5	54	F	Hematuria	Anterosuperior wall	36	PC	47	No
6	62	F	Hematuria	Right lateral wall	45	RC	48	No
7	40	M	Hematuria	Dome	35	PC	54	No
8	52	M	Hematuria	Right lateral wall	20	TURBT	24	No
9	14	M	Hematuria and odynuria	Anterior wall	45	PC	12	No
10	16	M	Hematuria and odynuria	Anterior wall	43	PC	48	No
11	70	F	Dysuria	Dome	20	PC	3	No
12	32	M	Urgent urination and odynuria	Dome	30	TURBT	6	No
13	43	F	Hematuria and odynuria	Anterior wall	43	PC	6	No
14	45	M	Hematuria and odynuria	Right lateral wall	30	TURBT	6	No

*PC, partial cysectomy; RC, radical cysectomy; TURBT, transurethral resection of bladder tumor*.

### Treatment and Follow-Up

All the patients underwent minimally invasive surgery. Eleven (78.6%) of them were treated with partial cystectomy, three (21.4%) patients were treated with TURBT, and one (7.1%) patient was treated with radical cystectomy (RC). Of the 3 patients who initially underwent TURBT, 1 subsequently underwent TURBT again. The mean follow-up was 43.9 ± 38 months (range 3–117). A female patient experienced recurrence within half a month and then underwent further TURBT and had no recurrence within 6 months. The symptoms of the other patients were significantly relieved after surgery, without local recurrence or distant metastasis. The patients had neither local recurrence nor distant metastasis by cystoscopy or CT scan. The treatment and follow-up outcomes are presented in Table 1.

### Histopathological Features

**Figure 1:**
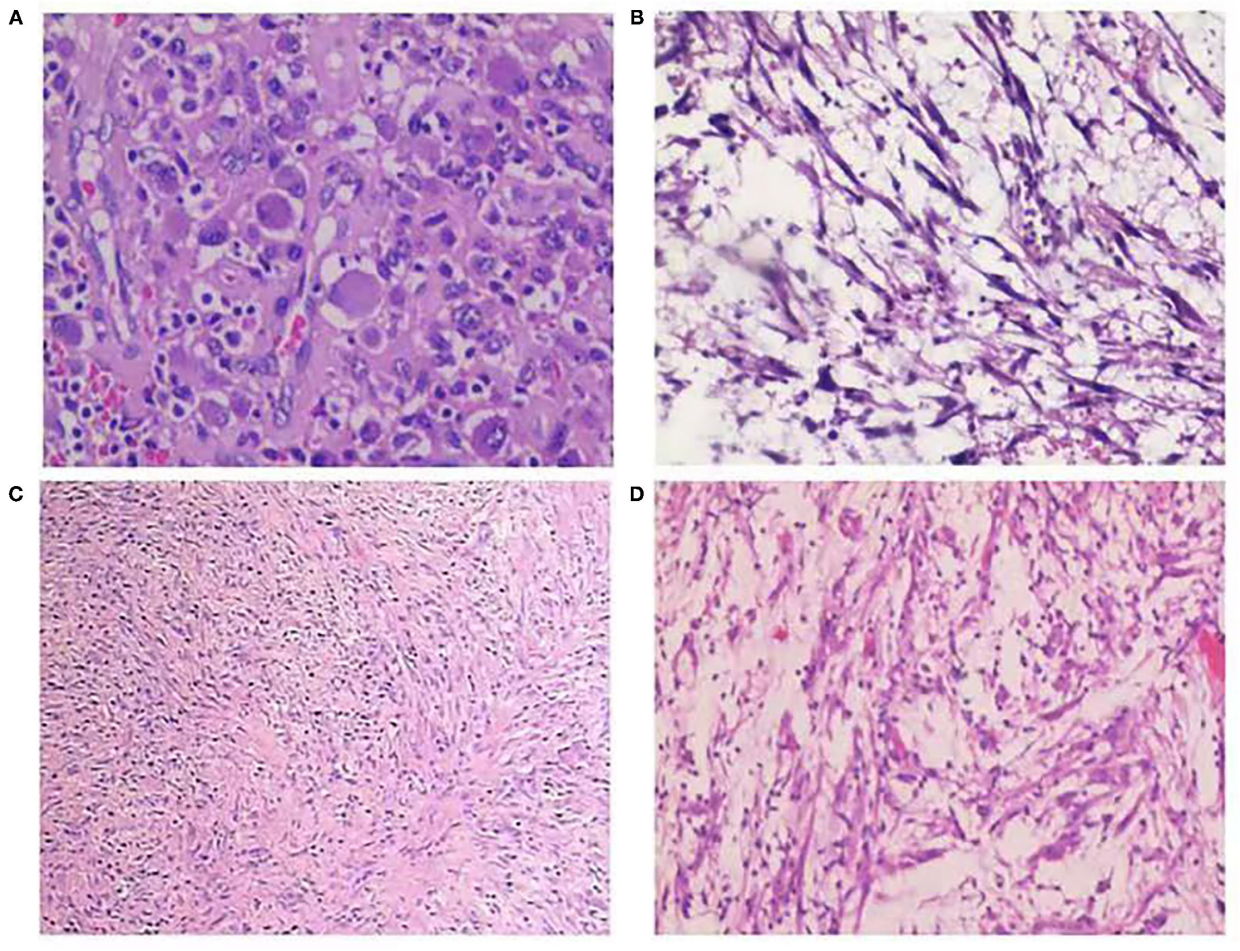
**(A)** Myxoid/vascular type [hematoxylin and eosin (H&E) stain, ×200]. **(B)** Compact spindle cell type (H&E stain, ×100). **(C)** Hypocellular fibrous type (H&E stain, ×100); **(D)** Myxoid/vascular pattern and hypocellular fibrous type (H&E stain, ×100).

Regarding the histopathological type, three (21.4%) tumors were myxoid/vascular type (Figure 1A), eight (57.1%) tumors were compact spindle-cell type (Figure 1B), one (7.1%) tumor was a hypocellular fibrous type (Figure 1C), one (7.1%) tumor was both myxoid/vascular and compact spindle cell type, and one (7.1%) tumor was both myxoid/vascular and hypocellular fibrous type (Figure 1D). Among eight cases with compact spindle cell types, all were characterized by spindle cells arranged in bundles or layers. Of the 14 patients, necrosis was found in six (42.9%), atypia was found in three (24.4%), mild atypia was found in one (7.1%), mild–moderate atypia was found in two (14.3%), and moderate–severe atypia was found in one (7.1%). Half of the patients (50%) did not have any atypia. Of the 14 patients, large amounts of lymphocytes, neutrophils, plasma cells, and eosinophils were found in 11 (78.6%), four (28.6%), two (14.3%), and one (7.1%), respectively. Only 1 case was noted to have two mitotic figures per 10 high-power fields. Of the 14 patients, tumor invasion to the muscularis propria was observed in 10 (71.4%), and invasion beyond the muscularis propria was observed in four (28.6%). The histopathological features are presented in Table 2.

**Table 2:** Histopathological features of the 14 cases of IMTUB.

**Case (*N*)**	**Histology pattern**		**Appearance of necrosis**	**Appearance** **of atypia**	**Appearance** **of pleomorphism**
1	Compact spindle cell type		–	Mild-moderate	–
2	Compact spindle cell type		+	Moderate-severe	–
3	Hypocellular fibrous type		–	+	–
4	Compact spindle cell type		–	–	–
5	Myxoid/vascular pattern and hypocellular fibrous type		+	Mild	–
6	Compact spindle cell type		–	Mild-moderate	–
7	Compact spindle cell type		–	–	–
8	Compact spindle cell and myxoid/vascular type		+	+	–
9	Myxoid/vascular type		–	–	–
10	Myxoid/vascular type		+	–	–
11	Compact spindle cell type		+	–	–
12	Compact spindle cell type		–	–	–
13	Compact spindle cell type		+	–	–
14	Myxoid/vascular type		–	+	–
**Case (** ***N*** **)**	**Appearance of abnormal mitosis**	**Mitotic figure/10 HPFs**	**Tumor invasion into MP**	**Tumor invasion beyond MP**	**Inflammatory cell type**
1	–	–	+	+	Lymphocytes, eosinophils
2	–	–	+	+	Lymphocyte
3	–	–	–	–	Lymphocyte
4	–	–	+	–	NA
5	–	2	+	+	Lymphocyte, neutrophil
6	–	–	+	–	Lymphocyte
7	–	–	–	–	NA
8	–	–	+	–	NA
9	–	–	–	–	Lymphocyte, neutrophil
10	–	–	+	+	Lymphocyte, neutrophil
11	–	–	+	–	Lymphocyte
12	–	–	+	–	Lymphocyte
13	–	–	+	–	Lymphocyte and plasma cells
14	–	–	–	–	Lymphocyte, neutrophil and plasma cells

*MP, muscularis propria; HPFs, high power fields; NA, not available; +, positive; –, negative*.

### Immunohistochemistry Features

**Figure 2:**
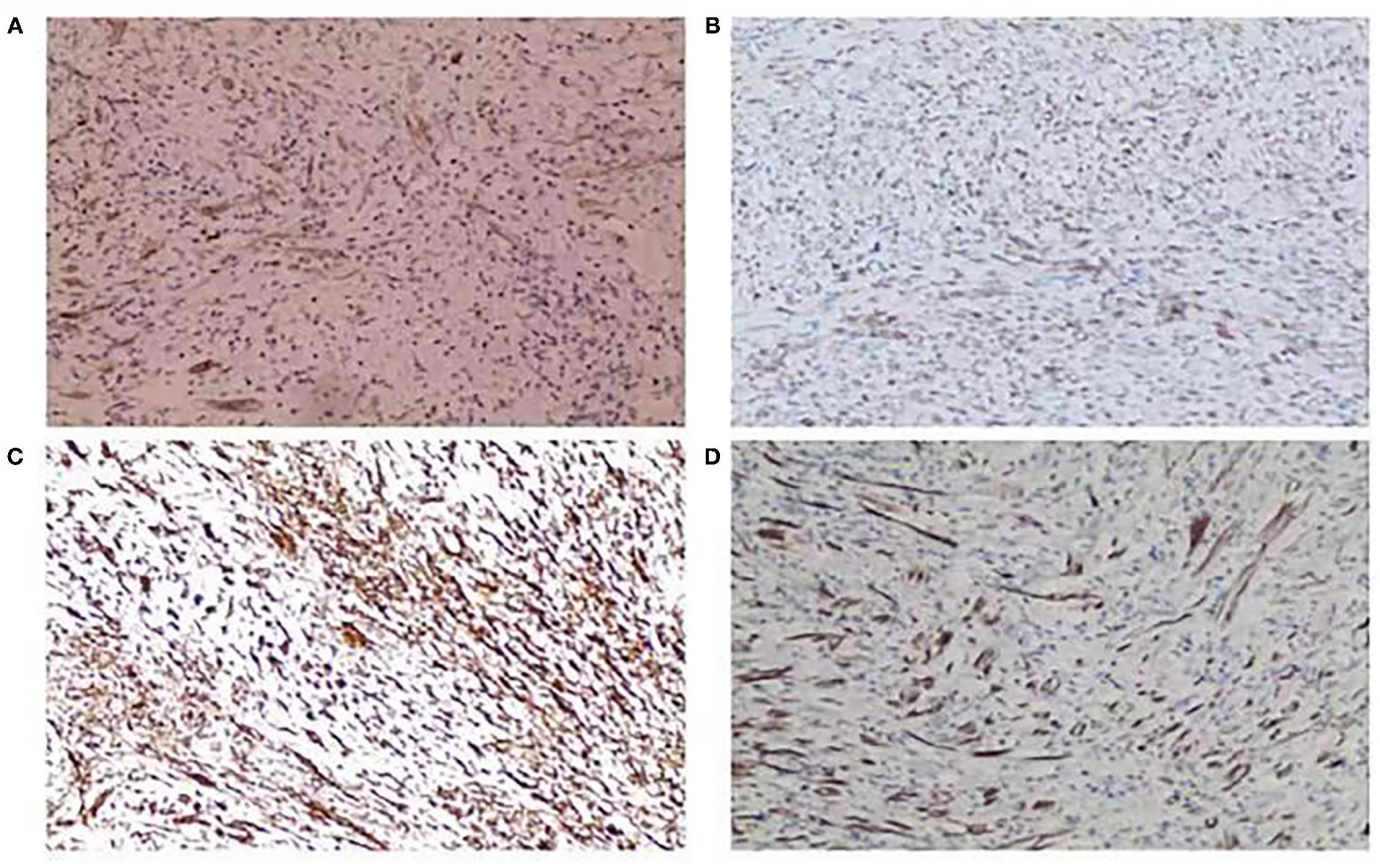
**(A)** Positive anaplastic lymphoma kinase (ALK) staining (ALK stain, ×100). **(B)** Positive smooth muscle actin (SMA) staining (SMA stain, ×100). **(C)** Positive vimentin staining (vimentin stain, ×100). **(D)** Positive cytokeratin (CK) staining (CK stain, ×100).

Concerning immunohistochemistry outcomes, tissues from all the patients were stained differently. Anaplastic lymphoma kinase (ALK) staining was positive in six (75%) of eight patients (Figure 2A). Smooth muscle actin was positive in all the cases (11 focal, three diffuse) (Figure 2B). The mean Ki-67 level was 14 ± 8.2% (range 1–30%). S-100 was positive in four (28.6%) of the 14 cases. Vimentin (Figure 2C), cytokeratin (Figure 2D), desmin, CD117, CD68, CD34, and HMB-45 were positive in nine (100.0%) of nine, nine (64.3%) of 14, five (50%) of 10, one (14.3%) of seven, eight (100%) of, eight (66.7%) of the, and one (25.0%) of four cases, respectively. The immunohistochemical staining outcomes are presented in Table 3.

**Table 3:** Immunohistochemical characteristics of the 14 cases of IMTUB.

**Case (*N*)**	**ALK**	**SMA**	**Ki-67**	**S100**	**Vimentin**	**CK**	**Desmin**	**CD117**	**CD68**	**CD34**	**HMB-45**
1	NT	+	30%	–	NT	+	–	NT	+	NT	–
2	NT	++	10%	–	+	–	–	–	+	–	–
3	NT	+	1%	–	NT	+	NT	–	NT	NT	NT
4	+	+	5%	–	+	–	–	–	+	+	NT
5	++	+	10%	+	++	+	–	+	+	+	NT
6	–	++	25%	–	++	+	+	–	+	+	+
7	NT	++	10%	–	NT	–	NT	NT	NT	–	NT
8	NT	+	25%	–	+	+	NT	NT	+	+	NT
9	–	+	15%	–	NT	+	+	NT	+	–	NT
10	+	+	15%	+	+++	–	–	–	NT	+	–
11	NT	+	10%	+	+	+	NT	–	NT	+	NT
12	+	+	10%	+	NT	+	+	NT	NT	+	NT
13	+	+	20%	–	+	–	+	NT	+	+	NT
14	+	+	10%	–	+	+	+	NT	NT	–	NT

*NT, not tested; –, negative; +, positve focally; ++, positive diffusely; +++, positive strongly*.

## Discussion

In 1939, IMT was first reported by Brunn as “myoma of the lung” (7, 9). In 1980, IMTUB, defined as a proliferative lesion of the submucosal stroma, was first proposed by Roth and showed low or uncertain malignant potential (9). In recent years, there have been different terminologies, such as inflammatory pseudotumor, pseudosarcomatous fibromyxoid tumor, pseudomalignant spindle-cell proliferation, and nodular fasciitis (6, 8). In 1994, IMT was defined as a neoplasm consisting of spindle cells characterized by myofibroblasts and a large number of associated inflammatory cells (3). An IMT can occur in the genitourinary system, but it is most common in the bladder and accounts for <1% of all bladder tumors (5, 7, 8). At present, the specific pathogenesis and etiology of IMT remain uncertain and might be connected with the following factors (4, 7, 10, 11): chronic inflammatory stimulation resulting from bacterial and viral microorganisms (mycobacteria, hepatitis B virus, Corynebacterium, Epstein–Barr virus, EBV, and human papillomavirus), history of bladder trauma or long-term use of hormone therapy, and rearrangements of the anaplastic lymphoma kinase (*ALK*) gene located on chromosome *2p23* (which occur in ~50% of IMTs). This prevalence of *ALK* rearrangements not only makes *ALK* a promising marker to diagnose and distinguish IMT from other tumors but also suggests that IMT may be neoplastic rather than postoperative spindle-cell nodule (PSCN). It represents a benign reactive myofibroblastic proliferation of the genitourinary tract within 3 months after instrumentation (8, 9). The inflammatory myofibroblastic tumor is a neoplasm of fibroblastic and myofibroblastic origin according to the 4th WHO classification. It is challenging to differentiate IMT from PSCN because of the overlap of morphology and immunohistochemistry; hence, clinical history can be helpful. Concerning genes, IMT may be more related to the clonal chromosomal rearrangement of *ALK* than PSCN. Leiomyosarcomas and sarcomatoid carcinomas usually lack *ALK* expression, especially when necrosis is present (2, 12, 13). A meta-analysis indicated that *ALK* played an important role in diagnosing and distinguishing IMTUB (14). The specificity and sensitivity were 0.99 (95% CI 0.67–1) and 0.86 (95% CI 0.58–0.96), respectively (14). The *Enterobacter cloacae*, a gram-negative bacillus that causes long-term chronic inflammatory stimulation of the bladder, was identified in urine cultures of two patients in this study. This bacterium has never been reported in previous literature.

**Figure 3:**
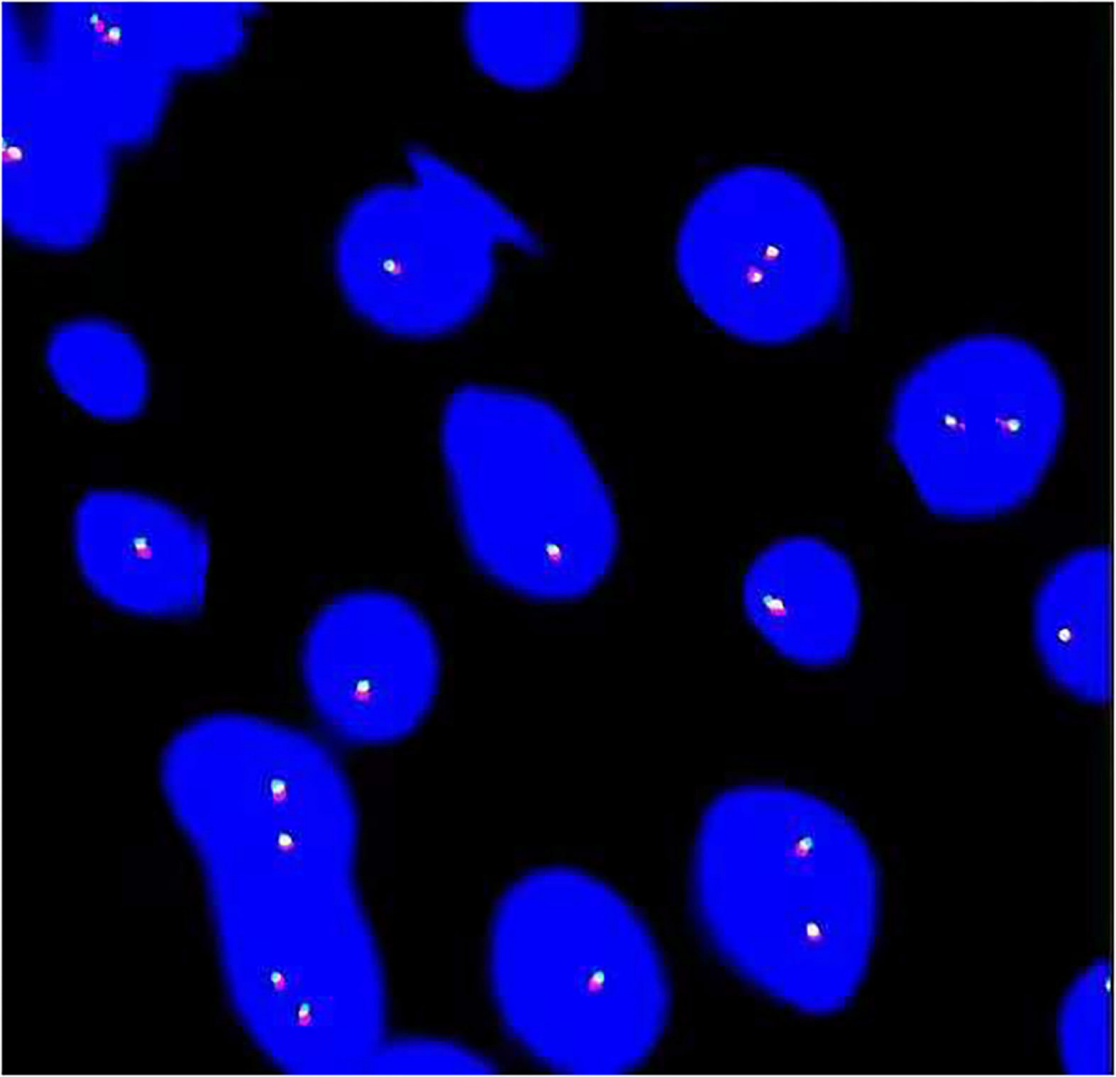
Fluorescence *in situ* hybridization (FISH) analysis revealed no arrangement of *ALK* in chromosome *2p23*.

Genomic rearrangements involving the *ALK* gene fusion with different partners, such as *TPM3, TPM4, CLTC, CARS, ATIC, SEC31L1, PPFIBP, DCTN1, EML4, PRKAR1A, LMNA, TFG, FN1*, and *HNRNPA1*, in IMTUB, have been described (8, 15–17). In recent years, novel *FN1-ALK* and *HNRNPA1-ALK* gene fusions have been discovered, which may suggest new targeted therapies in the future (15–18). However, Acosta, A.M et al. (16) reported that *FN1-ALK* gene fusion was characteristic of pseudosarcomatous myofibroblastic proliferation, which is a novel terminology for a tumor with significant clinicopathologic differences from IMT. Pseudosarcomatous myofibroblastic proliferation, with recurrence of 10–25% and without risk of metastasis, has a better prognosis than IMT. To further clarify the diagnosis, a FISH examination with the *ALK1* break-apart probe was carried out on cases 9 and 14. It confirmed no rearrangements of chromosome *2p23* (Figure 3). We advised the other patients to return to the hospital for the FISH test, but none of them complied.

The most common initial presentation of IMTUB is painless gross hematuria, but frequent urination, dysuria, abdominal/pelvic pain, and obstruction symptoms also exist (5, 8, 10, 11). Severe anemia can also develop. In a recent review of children with IMTUB from 42 studies around the world, the mean age was 7.5 years (range 2–15) (13). Forty-one of the children underwent surgery to remove the tumor, and one was treated with a 2-week course of anti-inflammatory therapy (13). Local recurrence was suspected in only one asymptomatic patient who was found to have a residual mass in the trigone during follow-up (13). Li et al. reported data from eight children (11). The mean age was also 7.5 years (range 2.7–11.5) (11). Three of the patients had mean hemoglobin of 77 g/l due to severe hematuria (11). A systematic review (9) of 182 patients showed a mean age of 38.9 ± 16.6 years, and the majority of the patients were females. According to this review, the most common symptom was hematuria (71.9%), followed by dysuria (19.8%), increased urinary frequency (18.8%), lower abdominal pain (13.5%), and loin pain (2.1%) (9). A multicenter retrospective study presented nine patients with IMTUB with a mean age of 45.4 ± 22.8 years (range 11–78), and 55.6% of them were females (19). Eight (88.9%) of the patients presented with hematuria, four (44.4%) presented with dysuria, four (44.4%) presented with urinary frequency, and two (22.2%) presented with loin pain (19). Five (55.6%) of them showed anemia at presentation, with a mean hemoglobin level of 68 ± 13 g/l (range 48–80) (19). The ALK-positive IMTUB occurred more frequently in younger female patients than in patients in ALK-negative IMTUB, but there was no significant difference in prognosis between ALK-positive and ALK-negative IMTUB (9), which may be because IMTUB more commonly occurs in females than in males.

**Figure 4:**
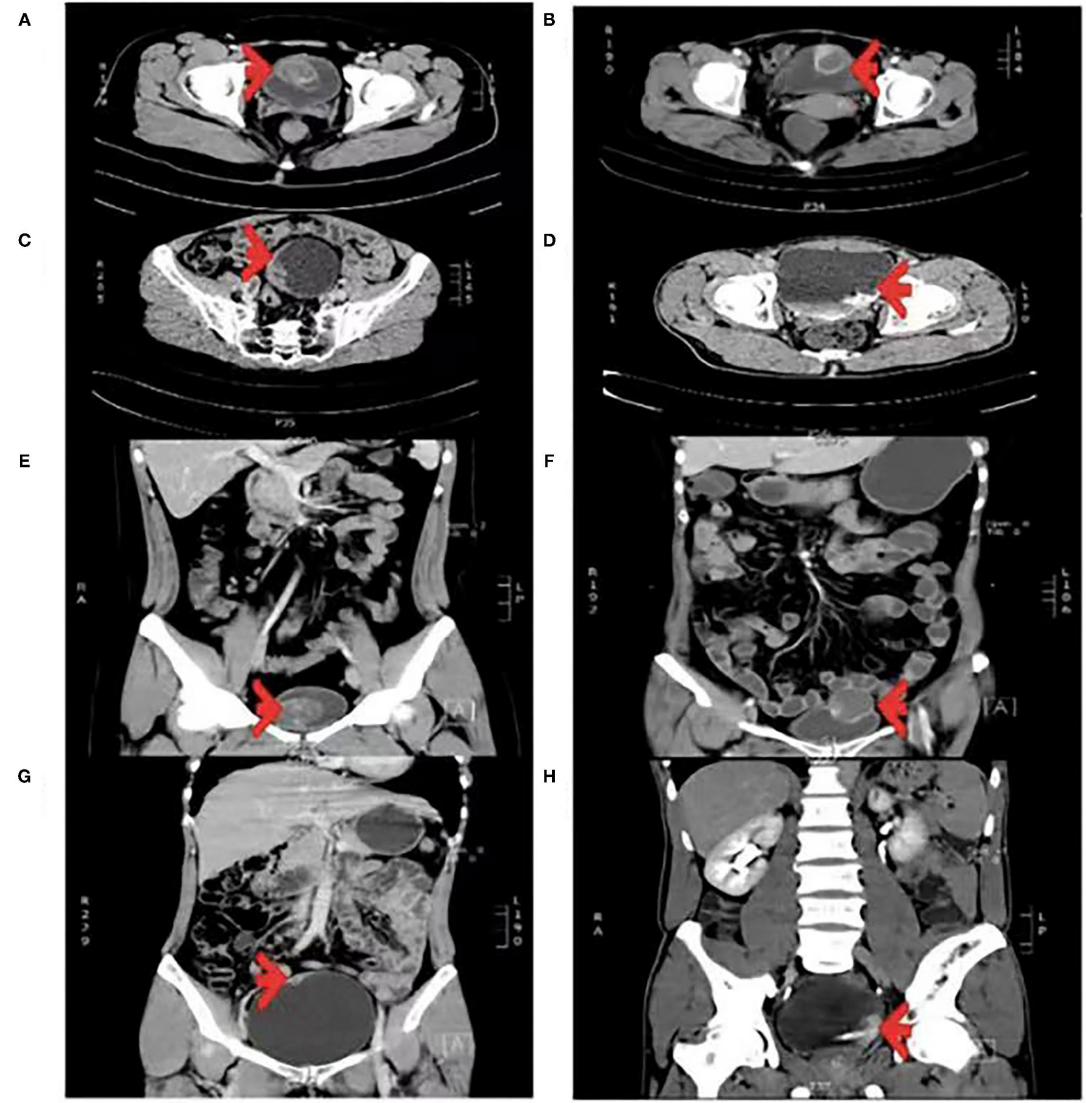
**(A–D)** Transverse enhanced CT scan. **(E–H)** Coronal enhanced CT scan. **(A,E)** Tumor with a size of 43 × 40 mm was located on the anterior wall of the bladder, papillary protrusion into the cavity with ring enhancement was present, and the CT value was 53 HU. **(B,F)** Tumor with a size of 36 × 33 mm was located on the anterior-superior wall of the bladder, exophytic growth with ring enhancement was present, and the CT value was 54 HU. **(C,G)** Tumor with a size of 48 × 12 mm was located on the right lateral wall of the bladder, papillary protrusion into the cavity was present, and the CT value was 59 HU. **(D,H)** Tumor with a size of 27 × 21 mm was located on the left lateral wall of the bladder, papillary protrusion into the cavity and suspected invasion to the left ureter were present, and the CT value was 59 HU.

**Figure 5:**
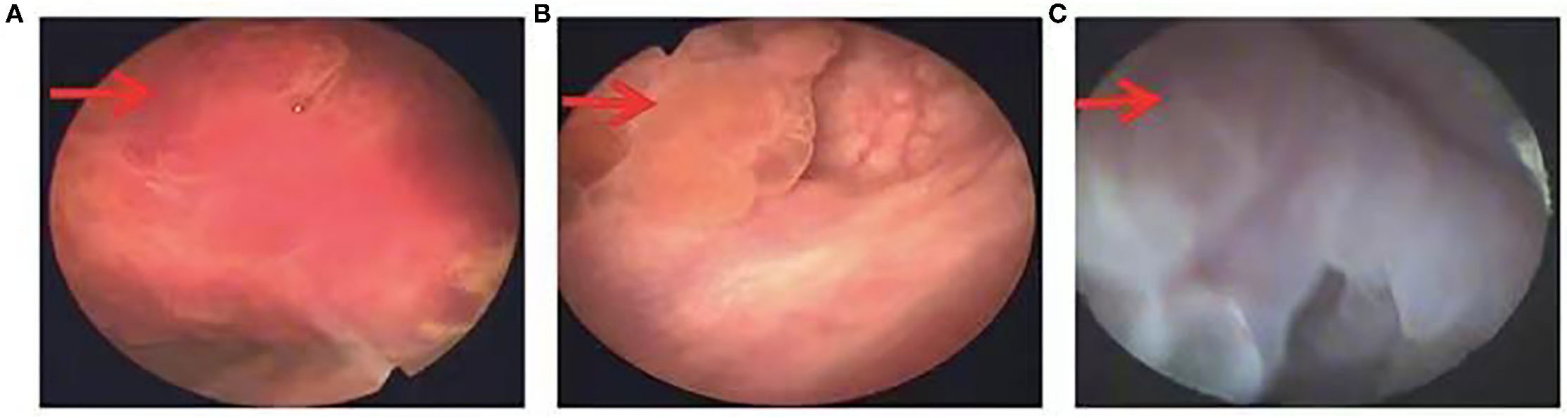
**(A–C)** Cystoscopy revealed cauliflower-like masses on the anterior wall, left lateral wall, and anterior wall of the bladder.

Radiographic examination usually indicates a space-occupying lesion with a lack of specificity in the bladder; therefore, it is difficult to differentiate IMTUB from bladder malignancy before surgery. However, Liang et al. (5, 6, 20) found that a primary finding of a lesion on the anterior wall of the bladder, with ring enhancement by contrast-enhanced CT, may be indicative of IMTUB. In our study, ring enhancement was observed on the anterior wall of the bladder in four (28.6%) out of the 14 cases and on the anterosuperior wall of the bladder in one (7.1%) on contrast-enhanced CT. The CT imaging features are described in Figure 4. Cystoscopy suggested a cauliflower-like mass in the bladder (Figure 5).

An IMT has a good prognosis and low risk of local recurrence and distant metastasis (6). The IMTUB shows a local recurrence rate of only 4% and a distant metastasis rate of lower than 5% after surgery (6, 11, 18). The preferred treatment choices for IMTUB mainly include TURBT, partial cystectomy, and/or radical cystectomy (5). Concerning the benign characteristics of IMTUB, bladder-sparing treatment modalities, such as TURBT and partial cystectomy, were better and did not increase the risk of recurrence, and resulted in fewer complications than radical cystectomy in one study (5). Partial cystectomy may be a better option, especially for patients with tumors invading the muscularis propria or the ureter. A recent systematic review of (9, 18) that included 182 cases of IMTUB showed that 60.8% of patients were treated with TURBT, 29.2% were treated with partial cystectomy, and 9.2% were treated with radical cystectomy. Some patients who were first treated by TURBT underwent a second TURBT (5.5%), partial cystectomy (17.8%), and radical cystectomy (1.4%) (9). The IMTUB was successfully treated with a selective cyclooxygenase-2 (COX-2) inhibitor, prednisolone, and combined with minimally invasive surgery in three teenagers (21, 22). Therefore, a good adjuvant strategy is to reduce tumor size and help preserve bladder function for large tumors with a selective COX-2 inhibitor combined with hormones. Wang et al. (23) reported that a 14-year-old patient diagnosed with IMTUB and distant metastasis was successfully cured with 5 months of preoperative adjuvant chemotherapy combined with radical cystectomy. Reinhart et al. also reported that neoadjuvant treatment with an ALK inhibitor helped enable complete tumor resection by partial cystectomy for large tumors with a size of 70 mm (18). Libby et al. (24) first reported that IMTUB caused local tumor recurrence and distant metastasis to the peritoneum and large intestines in a 61-year-old male who underwent radical cystectomy. Unfortunately, this patient died 3 weeks after this operation. The IMTUB invaded the peritoneum, ileocecal junction, greater omentum, appendix, and other tissues in two reported cases (12, 23). Encouragingly, there was no local recurrence or distant metastasis in either adolescent after 6–12 months (12, 23). The tumor may be characterized by aggressive growth if it extends to tissues outside the bladder, such as the peritoneum, greater omentum, and ileocecal region.

## Conclusion

An inflammatory myofibroblastic tumor of the urinary bladder (IMTUB) is a clinically rare tumor and has a good prognosis. The disease is mainly treated with TURBT and partial cystectomy to completely remove the tumor. It may be characterized by malignancy with aggressiveness if the tumor invades a distant site. Therefore, close follow-up is warranted. Based on this clinical retrospective study, more attention should be paid to IMTUB according to the features of CT and the pathology of IMTUB. It is really important to maintain patients' quality of life by preserving bladder function extensively, especially in young patients. Preoperative urine culture of *Enterobacter cloacae* in patients with IMTUB was an important discovery in our study. However, this finding may be incidental because of the limited number of cases. Therefore, further studies are needed to determine whether *Enterobacter cloacae* plays a role in IMTUB.

## Data Availability Statement

The original contributions presented in the study are included in the article/supplementary material, further inquiries can be directed to the corresponding author/s.

## Ethics Statement

The studies involving human participants were reviewed and approved by the Ethics Committee of the Second Xiangya Hospital of Central South University. Written informed consent to participate in this study was provided by the participants' legal guardian/next of kin. Written informed consent was obtained from the individual(s), and minor(s)' legal guardian/next of kin, for the publication of any potentially identifiable images or data included in this article.

## Author Contributions

RX and CC: conception and design. RX: administrative support. CC, MH, HH, SW, ML, JH, and HZ: provision of study materials or patients. CC, MH, HH, ML, and JH: collection and assembly of data. CC, MH, and HH: data analysis and interpretation. All authors: manuscript writing and final approval of the manuscript.

## Funding

This study was supported by the grant from Science and Technology Agency of Hunan Province (no. 2020JJ4820).

## Conflict of Interest

The authors declare that the research was conducted in the absence of any commercial or financial relationships that could be construed as a potential conflict of interest.

## Publisher's Note

All claims expressed in this article are solely those of the authors and do not necessarily represent those of their affiliated organizations, or those of the publisher, the editors and the reviewers. Any product that may be evaluated in this article, or claim that may be made by its manufacturer, is not guaranteed or endorsed by the publisher.

## Abbreviations

IMTUB: inflammatory myofibroblastic tumor of the urinary bladder
TURBT: transurethral resection of bladder tumor
PC: partial cystectomy
RC: radical cystectomy
ALK: anaplastic lymphoma kinase
IMT: inflammatory myofibroblastic tumor
SMA: smooth muscle actin
CK: cytokeratin
CT: computed tomography
NEUT%: neutrophil ratio
PCT: procalcitonin
CRP: C-reactive protein
ESR: erythrocyte sedimentation rate
EBV: Epstein–Barr virus.
